# Chicago Dental Society

**Published:** 1889-05

**Authors:** Louis Ottofy

**Affiliations:** Corresponding Secretary.


CHICAGO DENTAL SOCIETY.—At the annual meeting of the Chicago Dental Society, held on Tuesday evening, April 2, 1889, the following named were elected officers for the ensuing year: P. J. Kester, President; D. M. Cattell, First Vice-President; W. J. Martin, Second Vice-President; A. E. Baldwin, Secretary, Louis Ottofy, Corresponding Secretary; E. D. Swain, Treasurer; A. W. Harlan, Librarian. Member of the Executive Committee, J. Austin Dunn; Board of Censors, F. H. Gardiner, C. F. Hartt and S. S. Davis. Delegates to the International Dental Congress at Paris, France, September 1st to 8th, 1889, were appointed as follows: A. W. Harlan, (Secretary,) J. N. Crouse, T. W. Brophy, J. A. Swasey, P. J. Kester, W. W. Allport, A. E. Baldwin, Louis Ottofy, S. S. Davis, J. W. Wassall and W. B. Ames.

A report from Dr. Crouse showed the Dental Protective Association of the United States to be flourishing, and dentists throughout the United States are requested to become members of this Association.

LOUIS OTTOFY,
Corresponding Secretary.

